# Real-Time Analysis of Specific Protein-DNA Interactions with Surface Plasmon Resonance

**DOI:** 10.1155/2012/816032

**Published:** 2012-02-28

**Authors:** Markus Ritzefeld, Norbert Sewald

**Affiliations:** Department of Chemistry, Bielefeld University, P.O. Box 100131, 33501 Bielefeld, Germany

## Abstract

Several proteins, like transcription factors, bind to certain DNA sequences, thereby regulating biochemical pathways that determine the fate of the corresponding cell. Due to these key positions, it is indispensable to analyze protein-DNA interactions and to identify their mode of action. Surface plasmon resonance is a label-free method that facilitates the elucidation of real-time kinetics of biomolecular interactions. In this article, we focus on this biosensor-based method and provide a detailed guide how SPR can be utilized to study binding of proteins to oligonucleotides. After a description of the physical phenomenon and the instrumental realization including fiber-optic-based SPR and SPR imaging, we will continue with a survey of immobilization methods. Subsequently, we will focus on the optimization of the experiment, expose pitfalls, and introduce how data should be analyzed and published. Finally, we summarize several interesting publications of the last decades dealing with protein-DNA and RNA interaction analysis by SPR.

## 1. Introduction

DNA-protein interactions are involved in several biological processes like transcription, replication, DNA repair, or recombination. The specificity of such recognition processes originates from direct and indirect readout mechanisms. The variety of these mechanisms involves variations of the electrostatic potential due to groove narrowing or specific hydrogen bond donors and acceptors of the DNA helix that are recognized by a complementary set of amino acids [1]. 

Several methods have been developed to analyze DNA-protein interactions. Generally, they can be divided into two groups. Label-based methods require the ligation of the analyte and/or ligand with reporters like enzymes, fluorescent dyes, or radioisotopes. These labels possess the disadvantage that they can adulterate the results by interfering with the molecular interaction. Blocking the active binding site or affecting the conformation of the analyte can lead to false negatives. Moreover, unspecific background binding leading to false positives is another issue in these assays [2, 3]. In label-free approaches like atomic force microscopy-dynamic force spectroscopy experiments [4, 5], acoustic biosensors based on quartz crystal resonators [6], calorimetric biosensors [7], and surface plasmon resonance (SPR) inherently properties (e.g., mass) of the interacting molecules are measured. Therefore, these techniques avoid labeling steps and the disadvantages mentioned above. 

This article will focus on the most widely used label-free detection method: surface plasmon resonance. Although several suppliers like Biosensing Instrument Inc., Plexera LLC., or BioNavis Ltd. offer SPR-based instruments, Biacore (GE Healthcare) is by far the main supplier on the SPR market. In 2007, 89% of all publications dealing with surface plasmon resonance reported the use of a Biacore instrument [8]. We will, therefore, mainly place emphasis on Biacore instruments and nomenclature.

## 2. General Principle of SPR

### 2.1. SPR—The Physical Phenomenon

A beam of polarized light that propagates in a medium of high refractive index (e.g., a prism) is totally reflected, if it encounters an interface at a medium of low refractive index (*n*_2_) at a specific angle (Θ). This phenomenon is called total internal reflection (TIR). Although a total reflection occurs, the electromagnetic field component penetrates over a short distance into the medium of low refractive index. The resulting evanescent wave attenuates exponentially. If the interface is coated with a thin layer of metal (e.g., gold), a dip in the intensity of the polarized light will be visible (Kretschmann-Reather ATR configuration). Electrons are oscillating at the plasma frequency within the surface of the conductor. The quantization of this oscillation is called plasmon. The surface plasmons can couple with the photons of the polarized light, if the wavevector of the photon (*k*_*x*_) equals the wavevector of the surface plasmons (*k*_sp_). Coupling of both quasiparticles leads to an enhancement of the evanescent field amplitude (*E*). This phenomenon, called surface plasmon resonance, results in the observed dip of the light intensity. The wavevector of the plasmons depends on the refractive index of the conductor and the neighboring medium of low refractive index (*n*_2_). The wavevector of the photon depends on the wavelength of the polarized light and the angle of incidence (Θ). In conclusion, the refractive index *n*_2_ can be determined by measuring the intensity of the reflected light at different angles of incidence (Θ), if the wavelength of the polarized light and the refractive index of the conductor are both known [9, 10]. 

### 2.2. Using SPR for Interaction Analysis

**Figure 1:**
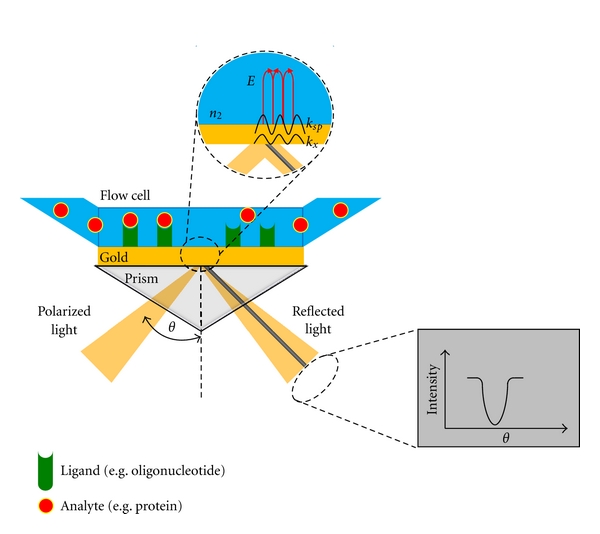
General principle of SPR. See text for details. *n*_2_ (refractive index of medium with lower refractive index), *E* (evanescent field amplitude), *k*_sp_ (wavevector of surface plasmons), *k*_*x*_ (wavevector of photon).

In most practical applications of SPR, the Kretschmann-Reather ATR method that was already described in the last section is used. In this setup, a thin metal film (typically around 50 nm thick gold layer) is evaporated onto the glass prism and kept in direct contact with the medium of lower refractive index (*n*_2_) [10, 11]. In order to evaluate the interactions of a protein with a nucleic acid that results in the formation of a protein-DNA complex, one of the two interaction partners has to be immobilized on the surface of the conductor (“ligand” in Figure 1) [12]. In most cases, a sensor chip with preimmobilized streptavidin is used to immobilize biotinylated oligonucleotides (more details concerning immobilization will be discussed below). The other interaction partner (e.g., the protein = “analyte” in Figure 1) is injected into the running buffer that passes the surface at a constant flow. In Biacore instruments, the resulting change in concentration of molecules at the gold surface due to the formation of the protein-DNA complex is measured in resonance units (RUs) and can be described according to (1):

(1) $$\begin{array}{c} \text{RU}=n\cdot \text{X}=\left[\text{RII}\cdot c\right]\cdot \text{X}=\left[{\left(\frac{\delta n}{\delta c}\right)}_{\text{ligand}}\cdot c\right]\cdot \text{X} \end{array}.$$

In this equation, *n* is the changing refractive index at the surface, X is a multiplier to convert *n* to RU, RII is the refractive index increment of the protein that is binding to the immobilized oligonucleotide, and *c* is the concentration of the protein. In general, 1000 RU correspond to a change in angle of 0.1°, or a protein concentration of 1 ng·mm^−2^ (alternatively 10 mg·mL^−1^) [13, 14]. One set of problems that is connected to the RII has to be mentioned when using the correlation of RU and protein concentration. The RII value of the molecules used is presumed to be in a range of ~0.18–0.19 mL·g^−1^. However, nonprotein molecules exhibit RII values beyond this range. In order to accurately perform an affinity ranking and correct stoichiometric measurements of small molecules the RU value has to be normalized for each measured compound [13]. Fortunately, the RII value is not important to get correct kinetic and thermodynamic results in simple protein-protein or protein-oligonucleotide interactions [15]. 

**Figure 2:**
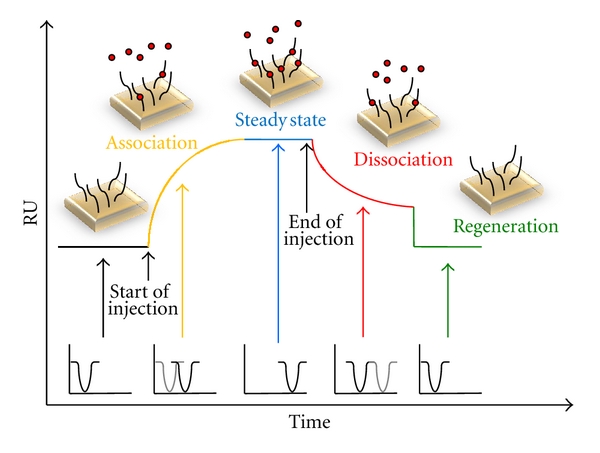
Typical shape of an SPR-sensogram. It can be divided into four phases: association phase, steady state or equilibrium phase, dissociation phase, and regeneration phase.

The typical shape of a sensogram that displays the change of the response units during the course of the experiment is shown in Figure 2. It can be divided into four different phases: association phase, steady-state or equilibrium phase, dissociation phase, and regeneration phase. The association phase starts with the injection of the analyte (e.g., protein). Due to the formation of a protein-DNA complex, the refractive index changes, resulting in a variation of the specific angle (Θ) where the dip in intensity of the reflected light is at its minimum. During the following equilibrium phase, association and dissociation of the complex occur at equal rates. Shortly, after the injection is terminated, dissociation of the analyte (e.g., protein) from the ligand (e.g., oligonucleotide) leads to a decrease in the response units. At any point of the dissociation phase, a regeneration buffer can be injected. It either contains a high salt concentration or detergents like SDS that release all remaining analyte molecules from the surface [12, 14]. After having finished the described cycle, another concentration of the analyte can be injected.

Every standard Biacore instrument is equipped with an integrated microfluidic cartridge (IFC) that forms four flow cells on the sensor chip and thereby enables the measurement of four different ligands at a time. In most applications, the first flow cell is used to substract response units resulting from unspecific interactions between the analyte (e.g., protein) and the chip surface or the analyte and flanking regions of the oligonucleotides' recognition sequence. This on-line referencing can be achieved by either keeping the first flow cell blank or by immobilizing an oligonucleotide that exhibits a random sequence [14, 16]. 

After having performed the experiment and referenced the results, the data is evaluated for example, using the BIA-evaluation software or Scrubber-2 (more aspects concerning data evaluation will be discussed below).

### 2.3. Fiber-Optic-Based SPR

**Figure 3:**
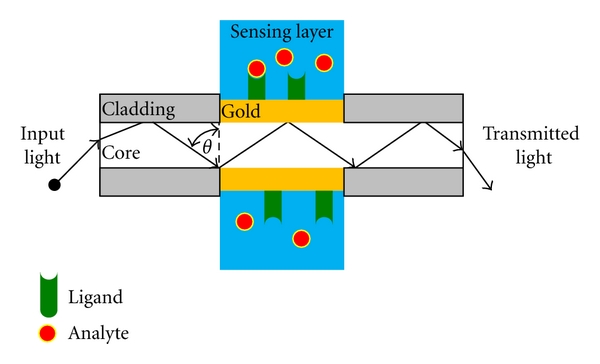
General principal of fiber-optic-based SPR (FO-SPR). See text for details.

The guidance of light inside an optical fiber is also based on total internal reflection (TIR). Therefore, the prism in the Kretschmann-Reather ATR setup can be replaced by the core of a fiber (cf.  Figure 3). In order to assemble a fiber-optic-based SPR (FO-SPR), the silicon cladding has to be removed in a small area of the optical fiber. The cleared core is surrounded with a thin metal coating and a dielectric sensing layer (cf.  Figure 3). Unlike prism-based SPR instruments, sensing is accomplished by changing the wavelength instead of the angle of incidence. Therefore, the resonance wavelength is measured instead of the resonance angle (Θ). Moreover, there is more than one reflection event. Due to the fact that the width of the SPR curve that affects the detection accuracy of the SPR instrument depends on the number of reflections, fiber parameters like length, sensing region, and fiber core diameter are crucial for the performance. To further change the sensitivity, detection accuracy and operating range of fiber optic-based SPRs, several modifications including a bimetallic coating, a tapered or u-shaped probe or the addition of dopants like GeO_2_ have been used. Although fiber-optic-based SPR instruments have some advantages like the simple and miniaturized setup that lacks moving parts or the possibility to assemble an inexpensive disposable sensor for medical or sterile tasks, the sensitivity is limited in comparison to the Kretschmann-Reather setup [10, 11]. 

### 2.4. SPR Imaging

**Figure 4:**
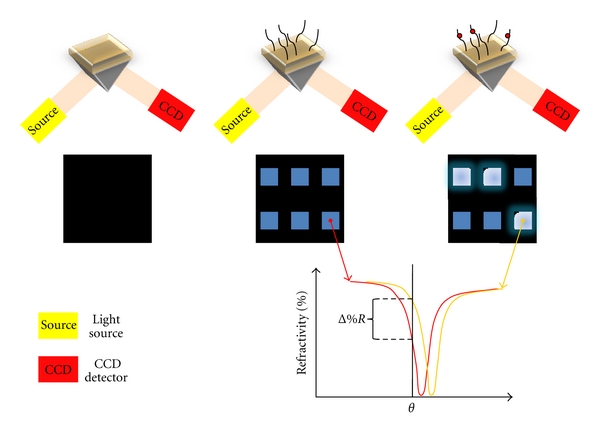
General principle of surface plasmon resonance imaging (SPRi). The reflected light of the whole array is detected using a CCD detector. Adsorption of a ligand on the sensor surface (middle) or the interaction between an analyte and the corresponding ligand (right) results in a shift of the SPR curve towards a higher angle (orange SPR curve). Due to the measurement restrictions (fixed wavelength and angle of incidence *θ*), binding is detected at every spot of the array simultaneously as a change in the reflectivity (Δ%*R*).

A combination of protein arrays and techniques like SPR would result in label-free alternatives to existing high-throughput methods that access kinetic data. However, the restriction that standard Biacore instruments are equipped with an IFC that forms four flow cells on the sensor chip complicates the expansion of SPR to high-throughput screening applications. Therefore, the sensitivity of SPR was combined with the features of imaging methods resulting in SPR imaging (SPRi) [17]. In SPRi, a coherent polarized light beam is expanded in order to cover a larger area of the sensing surface. The intensity of the reflected light is detected by a CCD (charge-coupled device) camera as image. In contrast to conventional SPR, the measurement is performed at a fixed wavelength and a fixed angle within a linear part of the SPR curve (cf.  Figure 4). Due to these confinements, changes in light intensity are proportional to alterations in the refractive index near the surface [18].

In an SPRi experiment, several images are taken during the course of time. For referencing, it is either possible to substract an image that was taken before the injection start or to do on-line referencing by keeping one spot of the array blank. Finally, a difference image of the array and a chart that displays the change in reflectivity for each probe during the course of time is obtained and can be evaluated further [19]. 

One problematic issue of SPRi is the intensity of the light source. Due to the proportional dependency of the signal strength on the incoming light intensity, laser beams are the preferred source. However, expanding the laser beam using optical elements results in an inhomogeneous illumination of the surface. Therefore, background correction is required that limits the resolution and sensitivity. New instruments with a scalable light source overcome the mentioned disadvantage by providing a more flexible illumination area [17].

## 3. Setting Up the Experiment

### 3.1. Immobilization

One advantage of label-free screens is that reporter groups that might interfere with the molecular interactions are unnecessary. However, in nearly all of the corresponding screening methods, also in the case of surface plasmon resonance, one of the interaction partners has to be immobilized. It is the most convenient and cost-effective way to immobilize short oligonucleotides as ligands on the surface to study protein-DNA interactions. If the unspecific interactions between the sensor chip and the analyte are too big or if the interaction between a single protein and several oligonucleotides has to be analyzed, it might be necessary to reverse the alignment of the experimental setup by immobilizing the protein on the sensor chip. In general, it is essential for the quality, validity, and reproducibility of the results to test and select the optimum immobilization method and assembly.

Due to the formation of stable sulfur-gold bonds, direct immobilization of thiol-containing ligands on a gold surface is possible [19]. One prominent example for the application of this method is atomic force microscopy. Proteins that lack cysteine residues can easily be modified using intein-mediated protein splicing combined with native chemical ligation, thus connecting a purification method with the ligation of a C-terminal cysteine [4]. 

However, proteins coming into contact with the metal can denature and undesired interactions with the surface are possible leading to inconsistent results [19, 20]. Therefore, in most SPR implementations, the gold surface is covered with hydroxyalkyl-thiols like 16-mercapto-hexadecan-1-ol thus creating a hydrophobic self-assembled monolayer (SAM) that prevents analytes and ligands to interact with the metal. Moreover, this layer serves as a functionalized structure that enables a further modification with carboxymethyl-modified dextran (e.g., Biacore's CM5-chip) [20]. This hydrogel facilitates the application of several immobilization strategies through linker molecules that can be attached covalently.

#### 3.1.1. Amine-Based Immobilization

**Figure 5:**
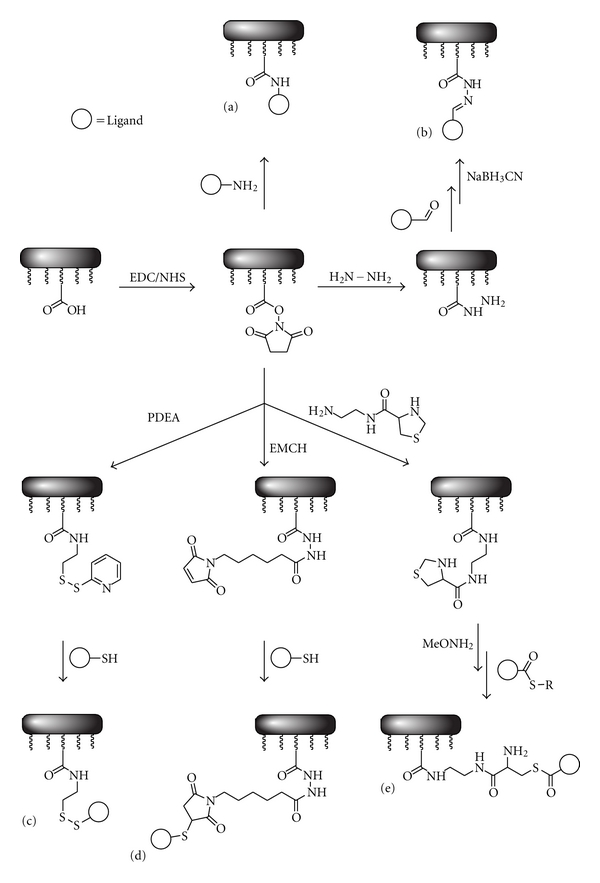
Examples of amine- and thiol-based immobilization methods using sensor chips coated with a carboxymethyl-modified dextran layer. (a): NHS/EDC-coupling of amine functionalized ligands, (b): immobilization of aldehyde funtionalized ligands using reductive ammination, (c): disulphide exchange, (d): ligation of thiol derivatives to maleimides, (e): native chemical ligation.

Biomolecules that exhibit free primary amines can be attached covalently to amine-reactive surfaces. In the case of sensor chips functionalized with a carboxymethyl-modified dextran layer (e.g., Biacore's CM5), the carboxyl groups can be activated by *N*-ethyl-*N′*-(dimethylaminopropyl)-carbodiimide (EDC), and N-hydroxyl succinimide (NHS). The resulting active ester reacts readily with free primary amines (cf.  Figure 5(a)) [21]. 

Sensor chips functionalized with aldehyde-terminated SAMs can be reacted with amine-modified oligonucleotides and proteins in aqueous buffer at basic pH, respectively. In both cases, the resulting imines are subsequently reduced using NaBH_3_CN [22, 23]. A similar approach starts with coupling of hydrazine to the carboxymethyl-modified dextran layer using EDC/NHS coupling followed by the addition of ligands with aldehyde substituents (cf.  Figure 5(b)) [24]. 

One disadvantage of these methods is the abundance of reactive amines in proteins. Due to the fact that several proteins contain more than one lysine residue, coupling of amines will lead to a heterogeneous population of ligands with random orientation and eventually random accessibility of the interaction site. 

#### 3.1.2. Thiol-Based Immobilization

Due to the lower abundance of cysteine residues in proteins, thiol-reactive surfaces combine the advantages of sensor chips equipped with SAMs with a more specific immobilization mechanism in comparison to the amine-based coupling reactions. Gold surfaces coated with carboxymethyl-modified dextran can be derivatized with sulfhydryl-reactive reagents like pyridinyldithioethanamine (PDEA) using the EDC/NHS coupling described above. Addition of a cysteine-containing protein results in the formation of a reversible disulphide linkage between the ethanamine and the ligand (cf.  Figure 5(c)). Residual free thiol groups are subsequently saturated using PDEA [25]. 

A second method based on thiol-reactive sensor chips involves maleimide-modified surfaces. Therefore, *N*-[*ε*-maleimidocaproic acid]-hydrazide (EMCH) is coupled to a chip coated with carboxymethyl dextran (e.g., CM5) using EDC/NHS (cf.  Figure 5(d)). Another similar approach involves coupling of ethylenediamine using EDC/NHS followed by the addition of *N*-[*γ*-maleimidobutyryloxy]sulfo-succinimide ester (sulfo-GMBS) [26]. Direct immobilization of maleimide derivatives on a gold surface without using a carboxymethyl dextran-coated sensor chip can be achieved by addition of maleimide-ethylene glycol-terminated disulfide (MEG) to a bare gold surface or sulfosuccinimidyl 4-(*N*-maleimidomethyl)-cyclohexane-1-carboxylate (SSMCC) to a gold surface coated with Fmoc-protected 11-mercaptoundecylamine (MUAM). In both cases, thiolated oligonucleotides (SH-DNA) have been successfully immobilized [27, 28]. 

A third method based on thiol-reactive sensor chips involves native chemical ligation. Coupling of thiazolidine-4-carboxylic acid (2-aminoethyl) amide to a CM5 sensor chip using EDC/NHS followed by deprotection of the thiazolidine ring with methoxyamine hydrochloride results in a free immobilized cysteine residue that readily undergoes native chemical ligation with peptide thioesters or expressed protein thioesters (cf.  Figure 5(e)) [29]. 

If commercially obtained thiol-functionalized DNA is used without further purification in any of the immobilization procedures described above, it has to be taken into consideration that the diversity of techniques for synthesis and purification can lead to a variety of impurities still present in the sample. Compounds like dithiothreitol (DTT) used to cleave the dimethoxytrityl protection group (DMT) can lead to a reduced amount of surface bound DNA [30].

#### 3.1.3. Enzyme Catalyzed Immobilization Reactions

In order to increase the specificity of the immobilization process, enzyme-catalyzed methods have been developed. 

O^6^-alkylguanine-DNA-alkyltransferase (hAGT) is an important DNA repair protein that removes alkyl groups from the O^6^-position of guanine. One pseudosubstrate of the hAGT, O^6^-benzylguanine is known to inhibit the transferase irreversibly. Hence, immobilizing an O^6^-benzylguanine–PEG-amino derivative via EDC/NHS chemistry followed by the addition of hAGT fused to a ligand of interest results in a covalent attachment of the fusion protein to the carboxymethyl dextran coated surface [31]. 

**Figure 6:**
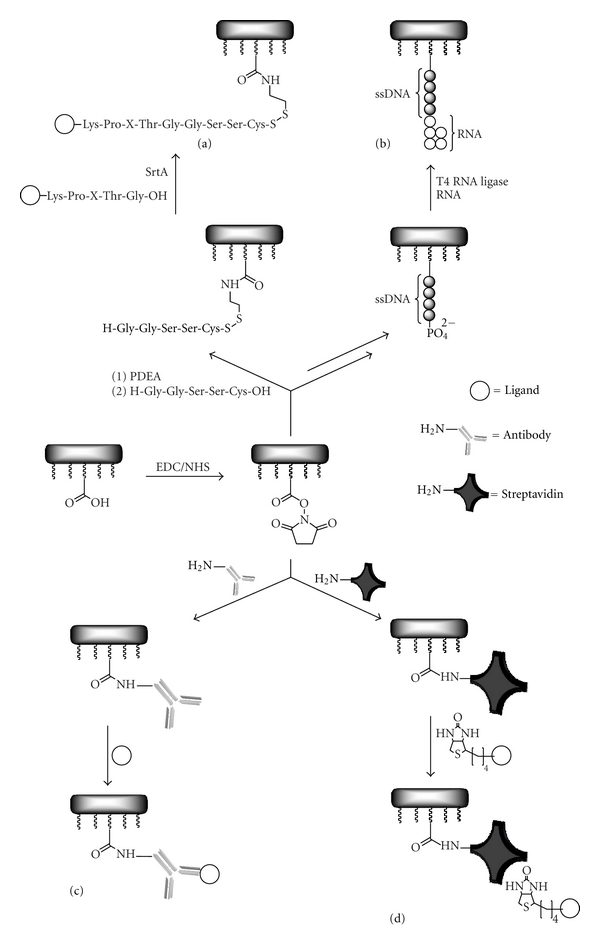
Examples of enzyme catalyzed- and affinity-based immobilization methods using sensor chips coated with a carboxymethyl-modified dextran layer. (a): Sortase A (SrtA) catalyzed ligation, (b): T4 RNA ligase catalyzed RNA immobilization, (c): antibody-facilitated immobilization, (d): streptavidin-biotin interaction.

A second approach is based on a transpeptidase reaction catalyzed by the sortase A (SrtA) from *Staphylococcus aureus*. SrtA cleaves between the threonin and the terminal glycine residues within the amino acid sequence Leu-Pro-X-Thr-Gly and links it to nucleophiles that exhibit an N-terminal glycine. Therefore, immobilizing a peptide with the amino acid sequence H-Gly-Gly-Ser-Ser-Cys-OH on a sensor chip surface using one of the thiol-coupling methods described above enables to attach proteins that contain the SrtA recognition sequence to the sensor chip surface by injecting the ligand of interest with the enzyme (cf.  Figure 6(a)) [32]. 

RNA oligonucleotides can be immobilized using T4 RNA ligase. Therefore, 5′-phosphate-terminated single-stranded DNA molecules are chemically immobilized on the sensor surface. T4 RNA ligase is then able to ligate RNA strands to the 5′-phosphate of the DNA. In order to eliminate the shielding effect of hairpin formation, the RNA has to start with eight adenosine bases at the 3′ end (cf.  Figure 6(b)) [33]. 

#### 3.1.4. Affinity Immobilization

Monoclonal or polyclonal antibodies capture the desired ligands with high selectivity and affinity. Moreover, they are easily regenerated at low pH, can be immobilized using most of the procedures presented here, and are commercially available for most antigens (cf.  Figure 6(c)) [26]. Beside all these advantages, the variable region (F(ab)_2_) of the antibody has to be exposed to the analyte. If physical adsorption or chemical coupling methods are used, only 20% of the antibodies have the right orientation to bind analytes properly [34]. Therefore, self-oriented immobilization methods involving proteins like protein A or G that specifically recognize the tail region (Fc) of the antibody have been developed. Hence, sensor chips coated with neutravidin-protein A complexes or protein G-DNA conjugates lead to an enhancement of the antibody/antigen binding ability [34, 35]. 

The biotin-streptavidin system exhibits the strongest noncovalent biological interaction known (*K*_*a*_ = 10^15^ M^−1^). Therefore, biotinylated ligands can be tethered to the surfaces of sensor chips functionalized with streptavidin (cf.  Figure 6(d)) [36]. The tetrameric protein can be immobilized on a standard sensor chip coated with carboxymethyl dextran (e.g., CM5) using EDC/NHS coupling [37]. Ready-to-use chips can also be purchased for example, from GE Healthcare (SA-chip). The biotin-streptavidin system is the method of choice for immobilizing nucleic acids [26].

In order to be applicable for immobilization, proteins can be biotinylated using NHS-active esters of biotin analogs that react with the *ε*-amino function of lysines. Further chemical biotinylation methods are based on *p-*diazobenzoylbiocytin that specifically labels tyrosine and histidine residues, or 3-(*N*-maleimidopropionyl)-biotin and iodoacetyl-LC-biotin that exclusively react with free thiols [38]. Although a substitution level of one biotin per ligand is recommended, the chemical methods described often result in multilabeled compounds, thus impairing the validity and significance of the SPR results [26]. Intein-mediated protein splicing combined with native chemical ligation using a cysteine biotin derivative is a more specific approach that overcomes this set of problems [39]. Moreover, the *Escherichia coli *(*E. coli*) biotin ligase (BirA) can be used to biotinylate site specifically a ligand fused to the recognition sequence of BirA [40].

Short biotinylated oligonucleotides, the most frequently used ligands for the analysis of protein-DNA interactions, can be readily purchased at every oligonucleotide supplier. The easiest way to obtain longer sequences is to use PCR with biotinylated primers. Another method is based on biotin-11-2′-deoxyuridine 5′-triphosphate (Biotin-11-UTP). The nucleotide can be incorporated into an oligonucleotide using nick translation or added as nontemplated nucleotide to the 3′-end of single and duplex DNA by the enzyme terminal deoxynucleotidyl transferase (TdT) [41, 42]. 

Although biotin interacts noncovalently with streptavidin, a reuse of the sensor chip is almost impossible due to the high affinity of the complex. Streptavidin binding peptide (SBP) is another interaction partner of streptavidin that possesses nanomolar affinity. Preparing a fusion protein consisting of the desired ligand and SBP facilitates moderate binding of the ligand and a complete removal from the surface using 1-min injections of 50 mM NaOH. An amino acid repeat of 5–15 glycine and serine residues between the ligand and the SBP enhances the flexibility and assures that the fusion protein is correctly folded [43].

One problem connected with streptavidin-coated chips is the occurrence of unspecific interactions with the surface. Electrostatic interactions between the negatively charged carboxymethyl dextran layer of the sensor chip and the protein used as analyte can significantly influence the SPR results. There are three different possible ways to overcome this limitation. First of all, changing the composition of the running buffer might help to reduce nonspecific binding (see below). Moreover, alternative usage of neutravidin which does not contain carbohydrate moieties minimizes nonspecific interactions [44]. The unglycosylated protein can be immobilized on a CM5-chip by EDC/NHS coupling [45]. A third method involves the usage of the commercial available hydrophobic sensor chip (HPA). The HPA-chip consists of a gold surface coated with an alkane-thiol layer. Phosphatidylcholine vesicles can be adsorbed onto the sensor chip and form a lipid monolayer. Addition of oligonucleotides tagged with a 3′-cholesterol group results in double-stranded DNA immobilized in a supported lipid monolayer that chemically and physically resembles a cell membrane surface and extensively reduces background interactions [46]. 

Another affinity immobilization method is based on nitrilotriacetic acid- (NTA-) modified sensor chips. Proteins labeled at the N- or C-terminus with oligo histidine (His) can be captured via Ni^2+^ NTA chelation. The choice of the utilized histidin-tag (e.g., hexa-His, deca-His, double-His tags) depends on the application of the SPR experiment. The surface can be regenerated conveniently by stripping the nickel using ethylenediaminetetraacetic acid (EDTA) solutions [47, 48].

Site-directed immobilization of a fusion protein consisting of a ligand of interest and the DNA binding domain of yeast Gal4 or the bacterial LexA is an affinity immobilization method based on protein-DNA interactions. Therefore, double-stranded oligonucleotides containing the recognition sequence of the DNA binding domain are spotted on poly(L-lysine-) coated gold chips [49].

### 3.2. Choosing the Right Conditions

#### 3.2.1. Analytes and Ligands

In general, the analyte and the ligand have to be chemically and conformationally homogenous to assure that the data is not corrupted by artifacts based on contaminations [50]. However, even crude samples like nuclear extracts that contain several different DNA binding proteins were successfully employed as analytes for oligonucleotides immobilized on a streptavidin-coated sensor chip. In order to specifically recognize the desired transcriptional activator and to amplify the corresponding signals, a primary antibody against the protein and a secondary antibody were added [51, 52]. Beside purity, concentration of the analyte is another very important aspect for reliable and high-quality results. The concentration of the analyte should cover a range from 0.1–10·*K_D_*. Moreover, at least five different concentrations should be used, including zero-concentration injections (blank injections, see below) [53]. 

Proteins used as ligands might lose their functionality in the course of time resulting in a signal drift. This phenomenon is, for example, known for proteases [54, 55]. By using stabilized mutants or by chemically crosslinking the protein, the stability for the HIV-1 protease was increased [54]. Although, the kinetic property of the crosslinked enzyme was similar to those of the native variant, the latter method has to be handled with precaution.

In the case of oligonucleotide ligands, the size of the molecule should correspond to the length of the DNA footprint, elongated by 3–6 extra base pairs as spacers on both sides [14]. Due to the fact that the surface plasmon wave decays evanescently approximately 200 nm into the solution, even oligonucleotides of considerable length can be used as ligands. The interactions of the transcription factor LEAFY from *Arabidopsis thaliana* to oligonucleotides that exhibit the sequences of the entire gene promoters *APETALA3* (2386 bp) and *APETALA3* (3050 bp) were successfully analyzed and the data quantitatively evaluated [56]. Also, oligonucleotides that possess hairpin conformation and contain a nick were directly immobilized on a gold surface. Ligation of the nick by DNA ligase of *E. coli* caused a change in the conformation from the hairpin structure to a rigid, linear double helix. The resulting change in the SPR dip shift was recorded using a noncommercial high-resolution SPR instrument [57].

#### 3.2.2. Referencing

As already mentioned above, the first flow cell is used to substract response units resulting from unspecific interactions. Therefore, the flow cell is either left blank or a reference compound is immobilized. If the cell is left blank, only unspecific interactions resulting from bulk refractive index changes, injection noise, baseline drift, or unspecific binding of the analyte to the surface are detected [50, 58]. In order to account for the refractive index changes caused by unspecific interactions between protein and DNA an oligonucleotide that exhibits a randomized sequence of the same length as the analyzed ligand should be immobilized [14]. If a protein is used as ligand, a compound with similar molecular weight and charge characteristics (e.g., a point mutant or denaturated sample that exhibits no affinity for the oligonucleotide) should be used as reference [59]. In both cases, the density of the ligand on the reference cell should approximate the density of the analyzed ligand [14, 59].

One referencing method that significantly improves the quality of the results is called double referencing. In doing so, signals collected from the reference cell are subtracted first of all. Afterwards, the average of the response units from injections of pure buffer is substracted from all obtained data sets [50, 53, 60].

#### 3.2.3. Mass Transport Limitation

Interactions of proteins with oligonucleotides can be very fast. If the kinetic rate constant *k_on_* is above 1·10^6^ M^−1^·s^−1^, it will be limited by the diffusion of the analyte to the immobilized ligand [61]. Due to the heterogeneity of the sensor chip surface, the transport of analyte through the microfluidic system and the nonstirred layer over the surface and the diffusion within the dextran matrix must be considered [3, 62]. This phenomenon, called mass transport limitation, can be reduced by optimizing the experimental system. For kinetic measurements, the maximal response unit difference after injection of the analyte should not exceed *R*_max⁡_   = 100 RU [63]. The corresponding amount of ligand immobilized on the surface can be calculated using (2), where *M*_Ligand/Analyte_ is the corresponding molecular weight, *ν*_Ligand_ the valency of the ligand, and *R*_Ligand_ the amount of immobilized ligand in RU [53]:

(2) $$R_{\text{Ligand}}=\frac{R_{\max}\cdot M_{\text{Ligand}}\cdot \nu_{\text{Ligand}}}{M_{\text{Analyte}}}.$$

High flow rates (≥50 *μ*L·min^−1^) [14] also minimize mass transport effects. If the association and dissociation rate values for a given system are identical at different flow rates, no mass transfer limitation is to be expected [50]. Moreover, flow cell geometry influences mass transport [3]. New Biacore systems like the Biacore 3000 reduce these effects due to an optimized geometry [14]. 

If, however, mass transport still affects the kinetics after experimental optimization, a mass transport rate constant (*k_m_*) can be incorporated into the binding model [64]. The corresponding value can be determined using a modified kinetic model of the Biacore evaluation software. Typical values for 50–100 kDa proteins are of the order of 10^8^ RU·M^−1^·s^−1^ [65].

#### 3.2.4. Buffer and Regeneration Conditions

Typical buffers for DNA protein interaction analysis using surface plasmon resonance involve HBS-EP buffer (10 mM HEPES, 150 mM NaCl, 3 mM EDTA, 0.005% v/v polysorbate 20, pH 7.4), MES10 buffer (10 mM 2-(*N*-morpholino) ethanesulfonic acid, 100 mM NaCl, 1 mM EDTA, 0.005% v/v polysorbate 20, pH 6.25), or Tris10 buffer (10 mM Tris, 100 mM NaCl, 1 mM EDTA, 0.005% v/v polysorbate 20, pH 7.4) [58]. 

As already mentioned in Section 3.1.4, the negatively charged dextran matrix can lead to strong nonspecific interactions and reduce the data quality drastically [46]. Changing the composition of the buffer is a promising alternative to choosing a different sensor chip surface. Therefore, three different ingredients of the buffer are important. The salt concentration influences the protein-DNA interactions. A slight increase of the salt concentration decreases the overall binding affinity and reduces nonspecific recognition of oligonucleotides to an undetectable level. However, solutions with high ionic strength can be used to remove the protein from the oligonucleotide in order to regenerate the sensor chip for an additional experiment [58, 66, 67]. The nonionic polyoxyethylene surfactant polysorbate 20 (trade name: Tween 20) is widely employed in immunoassays, AFM, and SPR to reduce nonspecific adsorption of proteins due to hydrophobic interactions [68]. It should be taken into consideration that a small increase in binding affinity might be observed when the amount of surfactant is increased in the running buffer [58]. Adding 0.05% of the polyanionic carboxymethyl dextran is known to improve the signal-to-noise ratio in the case of protein-protein interactions by competing with the surface dextran [69, 70]. 

Regeneration of the surface by injecting a solution that disrupts the analyzed complex might be necessary in the case of very slow dissociation. For the optimization of the regeneration protocol, it has to be kept in mind that enough data points of the dissociation should be recorded to ensure an accurate fit of the dissociation part of the sensogram, afterwards. Moreover, the immobilized oligonucleotide must persist undamaged to facilitate additional measurements. Even the blank sensor chips endure only a certain range of chemicals and conditions. More details concerning their stability can be found in the suppliers' manuals. A few potential solutions should be scouted to determine the most applicable regeneration buffer, by applying the corresponding conditions to approximately five cycles of analyte binding and regeneration. An overlay of the responses of the analyte binding steps indicates if the immobilized ligand is stable during the regeneration procedure [26]. Typical regeneration conditions normally involve low (10 mM glycine-HCl) or high (1–100 mM NaOH) pH, high ionic strength (up to 5 M NaCl), or low concentrations of SDS (up to 0.5%) [26]. If oligonucleotides are used as ligands, an intense change of the pH results in an unfolding and denaturation of the DNA. Washing the surface with buffer that exhibits pH 7 and hybridization of the oligonucleotide by readdition of the complementary strand is necessary before initiating the next cycle [58]. Superior regeneration methods for DNA ligands involve the injection of 1 mM HCl, a mixture of 50 mM NaOH with 1 M NaCl, or low amounts of SDS [14, 26].

## 4. Analyze and Publish Data

A considerate evaluation of the data is as essential as performing an optimally planned experiment. Moreover, publishing the results according to several high-quality norms is another issue every biosensor user should be capable of and perform. In 2010, Rich and Myszka published a biosensor literature review that provides rules and guidelines concerning the preparation of publishable high-quality data [71]. Although the responses of the biosensor community varied, we definitely recommend every user to read “The Mighty Binders” and to reconsider ones' own way of dealing with SPR results critically [72, 73]. We will, therefore, recapitulate the main guidelines of Rich and Myszka and clarify them with basic knowledge about data evaluation in the following section.

### 4.1. Reproducibility of Measurements

As already mentioned in Section 3.2.1, the analyte concentration used should cover a range from 0.1–10·*K_D_*. Every measurement should be repeated and the average value used for evaluation. Moreover, different sample concentrations should be analyzed in a randomized fashion or high concentrations are analyzed at the beginning and additionally at the end of the experiment [50]. Taking into consideration that several factors like running buffer composition, regeneration conditions, immobilization procedures, and chemistry, potential impurities or even the analyte on its own might cause a degradation of the ligand or the sensor surface during the course of time, it is obvious, that the provisions mentioned above facilitate that the experimental setup is consistent. In order to prove this consistency and the reliability of the developed biosensor method, replicates of at least one series of measurements must be undertaken and an overlay of the results should be published [71]. By mischance, this fundamental scientific principle is neglected in biosensor publications very often [50, 71]. Even during the preparation of this review, most of the literature found did not contain replicates or included sensograms at all. 

One nice feature of GE Healthcares' instruments is that the Biacore Wizard included in the control software provides the programming of flexible applications. An automated routine of the developed cycle conveniently enables measurements over night or over the weekend and facilitates an accurate and consistent accomplishment of the planned steps for every analyte concentration [59].

### 4.2. Data Evaluation

Before extracting the kinetic and thermodynamic parameters, the responses measured in the reference cell are subtracted, unwanted parts of the sensogram (e.g., regeneration) are removed, the baseline of all response curves is adjusted to zero, and spikes are deleted. All of these operations and the following parameter extraction by curve fitting can be performed using the Biacore evaluation software (GE Healthcare) or other programs like Scrubber-2 (Myszka and collaborators; BioLogic Software) [12, 65, 74]. 

#### 4.2.1. Kinetic Analysis

To explain the kinetic principle behind a protein-DNA interaction, we exclusively focus on the 1 : 1 model or Langmuir isotherm. As already mentioned in Section 2.2, a sensogram consists of an association and a dissociation phase. The kinetics can be described by the scheme:

(3) $$\text{DNA}+\text{Protein}\overset{k_{a}}{\underset{k_{d}}{⇌}}\text{DNA}\cdot \text{Protein},$$

where *k*_*a*_ is the association rate constant and *k*_*d*_ the dissociation rate constant. The resulting rate of the complex formation at the time *t* can be expressed using the following differential equation:

(4) $$\begin{array}{cc} \frac{d\left(\text{DNA}\cdot \text{Protein}\right)}{dt} & =k_{a}\left[\text{DNA}\right]\left[\text{Protein}\right]\\ & \;-k_{d}\left[\text{DNA}\cdot \text{Protein}\right], \end{array}$$

where [DNA], [Protein], and [DNA·Protein] are the corresponding molar concentrations. There are three important ways to solve this equation: linearization, integration, and nonlinear regression (numerical integration). 

The first and archaic way to analyze the data is linearization. The appliance of this method for surface plasmon resonance has been described among others by Majka and Speck [14], O'Shannessy et al. [75], and Morton et al. [76]. Substituting [DNA] in (4) by [DNA]_0_ − [Protein], where [DNA]_0_ is the concentration of the ligand at *t* = 0, results in

(5) $$\begin{array}{cc} \frac{d\left(\text{DNA}\cdot \text{Protein}\right)}{dt} & =k_{a}\left({\left[\text{DNA}\right]}_{0}-\left[\text{Protein}\right]\right)\left[\text{Protein}\right]\\ & \;-k_{d}\left[\text{DNA}\cdot \text{Protein}\right]. \end{array}$$

The observed signal *R* approximates the formation of the protein-DNA complex and the maximum signal *R*_max⁡_ is proportional to the surface concentration of the pure oligonucleotide. Therefore, in the case of an SPR experiment, (5) can be written as

(6) $$\frac{d\left(R\right)}{dt}=k_{a}\left(R_{\max}-R\right)\cdot c-k_{d}\cdot R,$$

where *c* is the analyte (protein) concentration. Taking the natural logarithm of (6),

(7) $$\ln\frac{d\left(R\right)}{dt}=\ln\left(k_{a}\cdot R_{\max}\cdot c\right)-\left(k_{a}\cdot c+k_{d}\right)\cdot t$$

and substituting

(8) $${k_{s}=k}_{a}\cdot c+k_{d}$$

results in the final equation:

(9) $$\ln\frac{d\left(R\right)}{dt}=\ln\left(k_{a}\cdot R_{\max}\cdot c\right)-k_{s}\cdot t.$$

**Figure 7:**
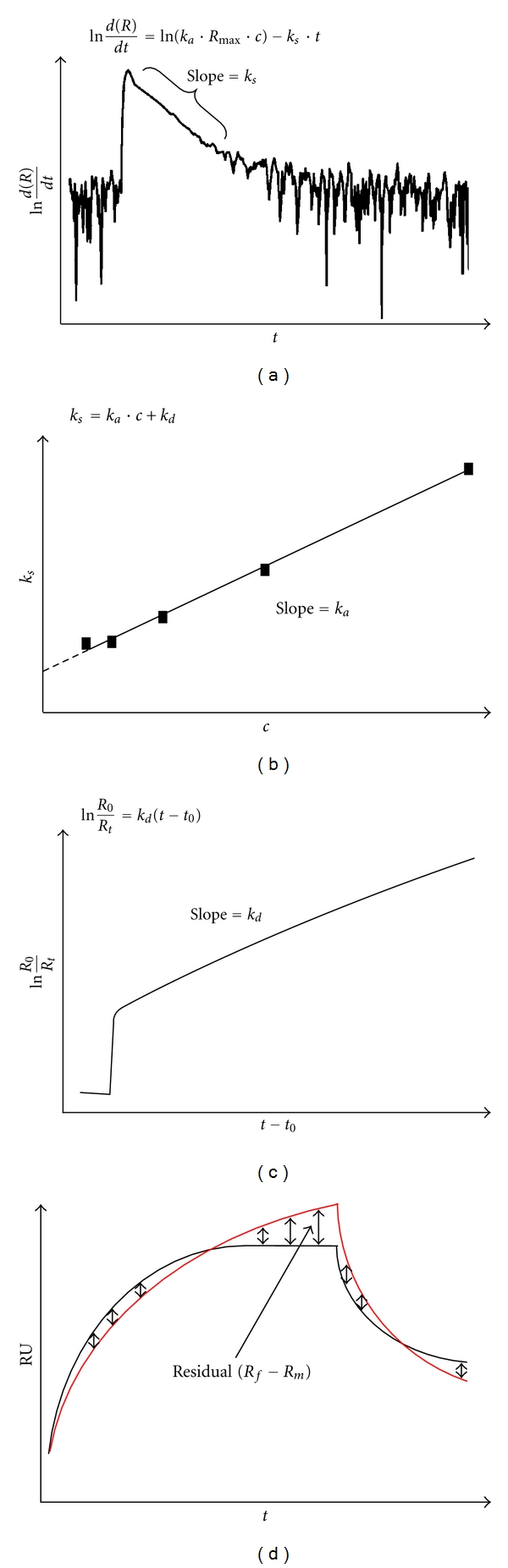
(a–c) Plots obtained during linearization of the association and dissociation phase of a sensogram using a 1 : 1 model. See text for details. Plotted data was taken from [14]. (d) Schematic overlay of a measured sensogram (black) and a calculated fit (red). Residuals (differences between the data points) are indicated by arrows.

Plotting of ln⁡⁡*d*(*R*)/*dt* versus *t* gives a linear function with the slope *k*_*s*_, if the results obtained for the analyzed system follow a 1 : 1 kinetic model (cf.  Figure 7(a)). The association rate constant can then be determined by plotting *k*_*s*_ versus *c*. According to (8), the slope of the corresponding linear function equals *k*_*a*_ (cf.  Figure 7(b)). 

The dissociation rate constant *k*_*d*_ has to be determined from the dissociation phase. Equation (10) describes the rate of this process:

(10) $$\frac{d\left(R\right)}{dt}=-k_{d}\cdot R.$$

The linearized form of (10) is

(11) $$\ln\frac{R_{0}}{R_{t}}=k_{d}\left(t-t_{0}\right),$$

where *R*_0_ is the response at *t*_0_. In analogy to (9), plotting of ln⁡*R*_0_/*R*_*t*_ versus (*t* − *t*_0_) gives a linear function with the slope *k*_*d*_, if the results obtained follow a 1 : 1 kinetic model (cf.  Figure 7(c)). 

One problem with linear transformations is that they adulterate the experimental error. The data points are assumed to be scattered in a Gaussian distribution around the regression line thereby exhibiting the same standard deviation. However, in most cases, transforming leads to an unequal distribution of the results. In conclusion, linear regression is less accurate [76, 77].

The second method to evaluate the SPR data involves direct analysis using the integrated form of the rate equation. Although, in contrast to linearization, errors in the derived parameters approximate the errors in the measured results, several biological systems cannot be described due to the fact that only simple bimolecular models can be evaluated using this method [75, 76].

The third way to analyze the data is based on nonlinear regression (numerical integration). Usage of this method to analyze data obtained by surface plasmon resonance is described in the BIAevaluation 3.0 Software Handbook [65] and has been reviewed among others by Morton et al. [76]. Moreover, the basic principle of numerical integration is explained on the webpage “curvefit.com—The complete guide to nonlinear regression” [77]. 

Using the Marquardt-Levenberg algorithm, kinetic models can be fitted to the data obtained. During this optimization process, the values of the corresponding kinetic variables (e.g., *k*_*a*_ and *k*_*d*_) of the fit are changed, until the lowest sum of the squared residuals *S* (cf.  Figure 7(d)) is reached. These residuals are calculated from the vertical distances between the measured sensogram and the calculated curve of the fit following (12):

(12) $$S=\overset{n}{\underset{l}{\sum }}{\left(R_{f}-R_{m}\right)}^{2},$$

where *R*_*f*_ is the fitted response value and *R*_*m*_ the measured one at a certain point. To determine the goodness of the fit, the *χ* ^2^-value is used:

(13) $$\chi^{2}=\frac{\sum_{1}{\left(R_{f}-R_{m}\right)}^{2}}{n-p}.$$

In (13), *n* is the number of data points and *p* the number of fitted parameters. The lower the *χ* ^2^-value, the better the corresponding fit. Acceptable values are *χ* ^2^ ≤ 10. Plotting the residual versus the *x*-values (in the case of SPR *x* = time) is another possibility to visualize the goodness of the fit. Besides, sensograms should include an overlay of the fit to further demonstrate the congruency. In order to resolve even the last doubts, SPR curves should be simulated using the model and the corresponding kinetic constants derived from the fit. Only if all of these approaches match, an adequate fit is obtained [71].

Unlike linearization and integration, numerical integration offers the possibility to determine the rate constants with high accuracy by modeling a variety of complex kinetic mechanisms. It allows for the incorporation of effects like mass transport or rebinding that influence the data. In conclusion, it is the most generally applicable and robust method to extract kinetic parameters from SPR results.

#### 4.2.2. Steady-State Analysis

There are two different possibilities to extract the equilibrium dissociation constant *K*_*D*_. In the case of the first method, *K*_*D*_ can be calculated from the ratio of the association and dissociation constants derived from the kinetic analysis [60, 65]:

(14) $$K_{D}=\frac{k_{d}}{k_{a}}.$$

For the second approach, the response units in the equilibrium at different analyte concentrations are used. The resulting saturation curve is analyzed by nonlinear regression to extract the dissociation constant using (16):

(15) $$\begin{array}{cc} & \frac{d\left(R\right)}{dt}=k_{a}\left(R_{\max}-R_{\text{eq}}\right)\cdot c-k_{d}\cdot R_{\text{eq}}=0 \end{array}$$

(16) $$\begin{array}{cc} & \Leftrightarrow R_{\text{eq}}=\frac{\left(R_{\max}-R_{\text{eq}}\right)\cdot c}{c+k_{d}/k_{a}\;}=\frac{\left(R_{\max}-R_{\text{eq}}\right)\cdot c}{c+K_{D}}, \end{array}$$

where *R*_eq_ is the observed steady-state response and *c* the analyte concentration [78]. Calculating the equilibrium dissociation constant by nonlinear regression using the 1 : 1 model described by (16) or a bivalent binding model can be performed with software like GraphPad Prism (GraphPad Software) [77] or Origin (OriginLab) [78]. Although transforming the data into a linear form using the famous Scatchard plot is also possible, nonlinear regression is definitely the method of choice due to the already-mentioned disadvantages of linearization.

## 5. Applications

Surface plasmon resonance has been widely applied in the analysis of oligonucleotide interactions. The corresponding list ranges from the investigation of single nucleotide mismatches using hybridization experiments [79] and the research of triplexes consisting of dsDNA and peptide nucleic acid (PNA) [80] to the kinetic analysis of small molecule-nucleic acid interactions involving binding of heterocyclic diamidines to AT sequences [81]. 

Focusing on the applications connected with surface plasmon resonance in the field of protein-DNA and RNA interactions, several interesting implementations of the three SPR methods (SPR, FO-SPR, and SPRi) described above are summarized below. The publications are sorted chronologically and cover the period from 1991 until 2011. 

One of the first publications outlining the analysis of protein-DNA interactions by SPR was published by Jost et al. in 1991. The authors immobilized a biotinylated oligonucleotide consisting of 40 bp on a streptavidin-coated chip and investigated binding of the two nuclear repressor proteins R1 and R2 [82]. Two years later in 1993, Bondeson et al. determined the kinetic rate constants and the equilibrium dissociation constant of the lactose repressor-operator complex formation using the linearization approach described above [83]. Since then, SPR evolved to a powerful and meaningful method to study protein-DNA and RNA recognition.

### 5.1. Blaesing et al., Analysis of the DNA-Binding Domain of Escherichia coli DnaA Protein, 2000 [84]

An extensive analysis of the DNA-binding domain of the *E.coli's *DnaA protein was performed by Blaesing et al. DnaA binds specifically to consensus sequences in the chromosomal replication origin of the bacteria. The protein unwinds an AT-rich region at the left boundary. Other proteins required for the replication process can then bind to the *oriC*. First of all, Blaesing et al. optimized the SPR method by using low amounts of DNA (100 RU) and a high flow rate (100 *μ*L·min^−1^) to prevent mass transport effects. As ligand, biotinylated oligonucleotides consisting of 21 bp that contain the DnaA box sequence and flanking regions were immobilized on a streptavidin chip. As control, an oligonucleotide without a DnaA box was used. Moreover, a blank flow cell was used for referencing. Afterwards, purified DnaA and crude extract that contains the protein were used as analytes, respectively. The response differences and the elucidated equilibrium dissociation constants of both analytes were comparable. Then, binding of 36 different point mutants was investigated and the proteins divided into four different classes concerning their *K*_*D*_ values: mutants with reduced wild-type like binding, mutants with low dissociation rates, mutants with high association and dissociation rates, and mutants without DNA-binding activity. In summary, Blaesing et al. were able to identify and to distinguish the DNA-binding domain regions of DnaA that mediate sequence specificity from the ones that solely stabilize the DnaA box recognition.

### 5.2. Neylon et al., Interaction of the Escherichia Coli Replication Terminator Protein (Tus) with DNA: A Model Derived from DNA-Binding Studies of Mutant Proteins by Surface Plasmon Resonance, 2000 [85]

DNA replication termination protein Tus stops the process of chromosomal replication in the final stage in *E.coli *by forming a replication fork trap. The interaction between Tus and its recognition sequence (*TerB*) is the strongest known DNA-protein interaction (*K*_*D*_ = 3.4·10^−13^ M). Neylon et al. analyzed binding of Tus to 9 different oligonucleotides, including *TerB*, single-stranded DNA molecules and nonspecific oligonucleotides that do not contain the *TerB *sequence. Moreover, binding contributions of four different point mutants, one from inside and three from outside the core binding domain, were elucidated. The authors first of all optimized the salt concentration in the buffer. As expected from literature, a low ionic strength resulted in immeasurable fast association and immeasurable slow dissociation rates. Therefore, four different concentrations of KCl (250–400 mM) were investigated. The measurement of the four mutants that are characterized by binding constants differing by 4000 folds was feasible only at 250 mM KCl. Having optimized the measurement conditions, Neylon et al. successfully elucidated the kinetic and steady-state parameters and confirmed that Tus binds with very high affinity to *TerB* and nonspecifically to single-stranded oligonucleotides and DNA molecules that do not contain the *TerB* sequence. Furthermore, the authors proposed on the basis of their data that structural changes in Tus are involved in the binding process.

### 5.3. Tsoi and Yang, Kinetic Study of Various Binding Modes between Human DNA Polymerase *β* and Different DNA Substrates by Surface-Plasmon-Resonance Biosensor, 2000 [86]

In order to perform a detailed kinetic study of the proposed binding modes of DNA polymerases, polymerase *β* that lacks the 3′-5′-exonuclease activity was used as model system. Binding of the enzyme towards different DNA targets including single-stranded, blunt-end double-stranded, gapped and template-primer duplex DNA-containing several different mismatches was analyzed by SPR. The results first of all indicate that the polymerase recognizes single-stranded DNA molecules with a higher affinity than blunt-end double-stranded oligonucleotides. Using DNA template-primer duplexes, the authors were able to show that polymerase *β* binds in the template-primer region and in the single-stranded template overhang with a preference for the first one. The introduction of mismatches resulted in a decreasing affinity for the duplex region and an increase in the amount of protein bound to the overhanging single strand. The authors were able to show that polymerase *β* recognizes several kinds of oligonucleotides but exhibits a considerable preference for template-primer duplexes. Moreover, the enzyme is able to discriminate between matched and mismatched DNA.

### 5.4. Shumaker-Parry et al., Parallel, Quantitative Measurement of Protein Binding to a 120-Element Double-Stranded DNA Array in Real Time Using Surface Plasmon Resonance Microscopy, 2004 [67]

Shumaker-Parry et al. coated a gold surface with a SAM consisting of oligo(ethylene glycol)-terminated thiol (OEG) and biotin-terminated thiol (BAT). Using a commercial robotic microspotting system, the authors fabricated a 10·12 array by spotting 120 oligonucleotides of 100 and 77 bp in length. In this proof-of-principle experiment, Shumaker-Parry et al. only used two different DNA molecules: the binding site of the yeast transcription factor Gal4 and an oligonucleotide that lacks the Gal4 sequence. The authors used the latter as control spot for on-line referencing and analyzed the 120 spots simultaneously. In summary, Shumaker-Parry et al. reported a proof-of-principle for the usage of surface plasmon resonance imaging as high-throughput technique in the investigation of protein-DNA interactions.

### 5.5. Fang et al., Determination of Ribonuclease H Surface Enzyme Kinetics by Surface Plasmon Resonance Imaging and Surface Plasmon Fluorescence Spectroscopy, 2005 [87]

Fang et al. analyzed the kinetics of the hydrolysis of RNA-DNA heteroduplexes by ribonuclease H (RNase H) using surface plasmon resonance imaging and surface plasmon resonance fluorescence spectroscopy (SPFS). In SPFS, the enhanced field of the surface plasmon mode is used for the excitation of fluorophores attached to the immobilized ligand (here: the immobilized single stranded RNA). Using a fluorescence detection unit, a second readout mechanism facilitates an increasing sensitivity of the conventional SPR method.

**Figure 8:**
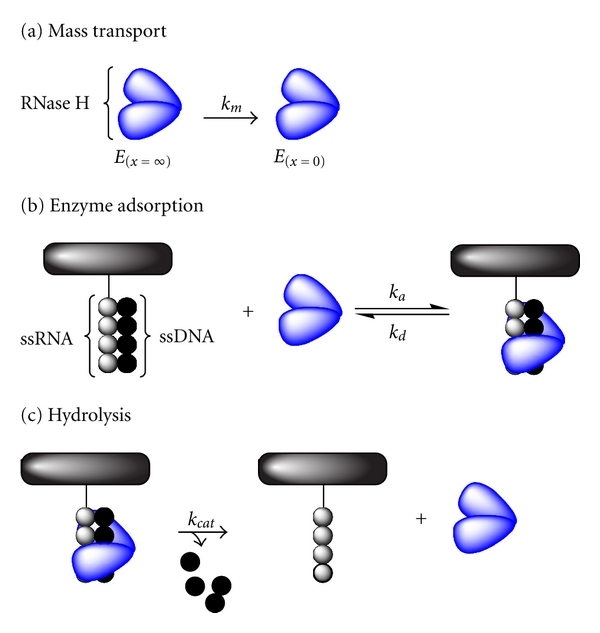
Illustration of the ribonuclease H reaction, involving (a) mass transport, (b) enzymatic adsorption, and (c) hydrolysis [89].

Having already shown that SPRi can be used to detect the ribonuclease H reaction in 2004 [88], the authors were interested in a complete characterization of the enzymatic reaction. Fabrication of the sensor array was performed using the MUAM/SSMCC method described above. To extract kinetic data from the sensograms, the authors created a model that includes the three rate constants enzyme adsorption (*k*_*a*_), enzyme desorption (*k*_*d*_), enzyme catalysis (*k*_cat_), and a dimensionless diffusion parameter (*β*). The corresponding reaction scheme can be written as

(17) $$E_{\left(x=\infty \right)}\overset{k_{m}}{\to }\;E_{\left(x=0\right)}+S\begin{array}{c} \overset{k_{a}}{\underset{k_{d}}{⇌}} \end{array}ES\overset{k_{\text{cat}}}{\to }\;S^{*}+E_{\left(x=0\right)},$$

where *E*_(*x*=*∞*)_ and *E*_(*x*=0)_ are the bulk and surface enzyme species, respectively, *k*_*m*_ is the corresponding mass transport coefficient, *S* the RNA-DNA heteroduplex, *ES* the enzyme-substrate complex, and S* the reaction product (single stranded DNA). A different illustration of the reaction scheme is presented in Figure 8. 

Using this reaction model, Fang et al. derived the following differential kinetic equation based on the relative surface coverages (*θ*_X_):

(18) $$\begin{array}{c} \theta_{S}+\theta_{ES}+\theta_{S^{*}}=1,\\ \frac{d\theta }{dt}=\frac{k_{a}\left({1-\theta }_{ES}-\theta_{S^{*}}\right){\cdot \left[E\right]}^{b}-\left(k_{d}+k_{\text{cat}}\right)\cdot \theta_{ES}}{1+\beta \cdot \left(1-\theta_{ES}-\theta_{S^{*}}\right)},\\ \frac{d\theta_{S^{*}}}{dt}=k_{\text{cat}}\cdot \theta_{ES}. \end{array}$$

By fitting the SPRi and SPFS datasets using (18) the values of the constants (*k*_*a*_, *k*_*d*_, *k*_cat_, and **β**) were calculated. In summary, the authors successfully examined and described the surface enzyme reaction of RNase H using surface plasmon resonance techniques.

### 5.6. Bouffartigues et al., Rapid Coupling of Surface Plasmon Resonance (SPR and SPRi) and ProteinChip Based Mass Spectrometry for the Identification of Proteins in Nucleoprotein Interactions, 2007 [90]

The authors compared a coupling approach of an LC-MS instrument to an SPR (Biacore 2000; GE Healthcare) and an SPRi system (SPRi-Plex; Genoptics). As evaluation system, binding of the bacterial nucleoid protein H-NS to high- and low-affinity sequences and the interaction between the integration host factor (IHF) and an oligonucleotide containing a single IHF binding site were analyzed. 

A direct coupling of the Biacore 2000 IFC to the reverse phase HPLC column of the LC-MS should facilitate the recovery and direct analysis of the analyte mixture. In the case of the SPRi, binding of the analyte mixture was first of all investigated using a standard protocol. Afterwards, the sensor array was incubated with the analyte mixture but not regenerated. The array was removed from the SPRi instrument, dried, and each spot independently treated with 1 *μ*L of the regeneration solution. Then, the regeneration solution of every spot was recovered and spotted onto a ProteinChip. After cocrystallization with a matrix, the ProteinChip was analyzed using surface-enhanced laser desorption/ionization mass spectrometry (SELDI). 

Bouffartigues et al. were able to show that a satisfactory recovery and identification was not possible in the case of the Biacore 2000. However, using the SPRi-based method, the authors successfully recovered and analyzed both proteins (H-NS and IHF) using mass spectrometry after having quantified the interactions.

### 5.7. Di Primo, Real Time Analysis of the RNAI-RNAII-Rop Complex by Surface Plasmon Resonance: From a Decaying Surface to a Standard Kinetic Analysis, 2008 [91]

RNA-RNA interactions between stem-loop structures are essential regulatory elements, for example, in prokaryotic organisms. In *E.coli, *two plasmid-encoded transcripts, RNAI and RNAII, regulate the replication of the plasmid ColE1. Interaction between the antisense RNA, RNAI, and the RNA primer, RNAII, prevents the formation of the RNA-DNA hybrid, necessary for the replication initiation. A protein (Rop), also encoded by the plasmid, stabilizes the loop-loop interactions. To study this system, Di Primo immobilized biotinylated RNAI on a streptavidin chip and saturated the chip with RNAII. Afterwards, increasing concentrations of Rop were injected. Instead of performing several cycles that include the injection of one concentration, followed by a regeneration step, Di Primo used kinetic titration experiments by injecting three concentrations of Rop sequentially. Evaluation of the reaction between Rop and the bimolecular RNA complex was accomplished by keeping the RNAII concentration in the injected flow at a high constant level. The results indicate that RNAII dissociates 110 times slower in the presence of Rop.

### 5.8. Pollet et al., Fiber Optic SPR Biosensing of DNA Hybridization and DNA-Protein Interactions, 2009 [92]

Although the first fiber-optic design was introduced by Jorgenson and Yee in 1993, only a small number of biosensing applications (especially concerning DNA-protein interactions) have been reported. Pollet et al. attached biotinylated ssDNA aptamers against human immunoglobulin E (hIgE). The authors confirmed the recognition specificity by repeating the experiment with hIgG as analyte. Moreover, the binding kinetics of the aptamer-hIgG interaction was determined by FO-SPR and the values confirmed by affinity studies on capillary electrophoresis and a prism-based SPR (Biacore 3000).

### 5.9. Pan et al., Double Recognition of Oligonucleotide and Protein in the Detection of DNA Methylation with Surface Plasmon Resonance Biosensors, 2010 [93]

Aberrant hypermethylation of CpG islands in promotor regions is a genome alteration frequently connected to human cancers. Hence, the methylation status is an important and promising target in diagnostics. However, detection methods involving methylation-sensitive restriction digestion or methylation-specific PCR are laborious and time consuming. Therefore, Pan et al. developed a double-recognition method based on SPR. The adenomatous polyposis coli (ACP) gene promotor 1A that exhibits 31 CpG islands and has been confirmed in several cancers was used as detection model. In the first step of the method, single-stranded genomic DNA was added to single-stranded biotinylated oligonucleotides that were immobilized on a streptavidin coated sensor chip and possess a certain sequence for a specific promotor region. In the second step, methyl-CpG binding domain (MBD) protein, that specifically binds symmetrically methylated oligonucleotides, was injected. To verify the specificity of the recognition process, poly(CGA) and methylated poly(_m_CGA) were immobilized on two other flow cells. The authors successfully verified the methylation of the corresponding promoters by SPR. Transferring this method to SPRi could result in high-throughput SPR sensors for methylation detection.

### 5.10. Šípová et al., A Dual Surface Plasmon Resonance Assay for the Determination of Ribonuclease H Activity, 2011 [94]

Šípová et al. developed an SPR-based method to determine the properties of antisense oligonucleotides using the endonuclease RNase H in an SPR experiment. Due to the ability of antisense molecules to hybridize sequence-specifically with single-stranded RNA like mRNA, injection of antisense strand into cells can result in the knockout of certain transcripts.

**Figure 9:**
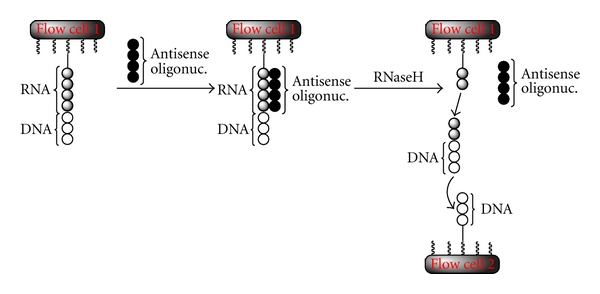
Addition of antisense RNA to the chimeric oligonucleotide consisting of DNA and RNA, results in the hybridization of the antisense strand and its complementary RNA sequence. RNase H only recognizes RNA-DNA heteroduplexes and cleaves the corresponding RNA strand. The resulting chimeric fragments end up in the next flow cell (flow cell 2). Due to the complementarity between the immobilized single stranded DNA in flow cell 2 and the DNA of the chimeric oligonucleotide fragments, both strands hybridize and induce a response in flow cell 2.

In the first step of the dual assay, biotinylated chimeric oligonucleotides that consist of an RNA sequence and a short DNA strand ligated to its 3′-end were immobilized on a streptavidin sensor chip (cf.  Figure 9 left). An antisense oligonucleotide, complementary to the ribonucleotide sequence of the immobilized molecule, was injected and a heteroduplex was formed (cf.  Figure 9 middle). In the following step, RNase H was added. The enzyme recognizes the heteroduplex consisting of the RNA sequence and the antisense strand and cleaves the RNA part. The produced fragments were released into the solution and hybridize with complementary oligonucleotides immobilized in the following flow cell (cf.  Figure 9 right). The DNA fragment of the chimeric DNA molecule was necessary, to enhance the SPR response in the first flow cell and to facilitate the specific hybridization with the immobilized ligands in the second flow cell. This method has the potential to screen the properties of antisense oligonucleotides containing chemical modifications.

### 5.11. Pollet et al., Real-Time Monitoring of Solid-Phase PCR Using Fiber-Optic SPR, 2011 [95]

**Figure 10:**
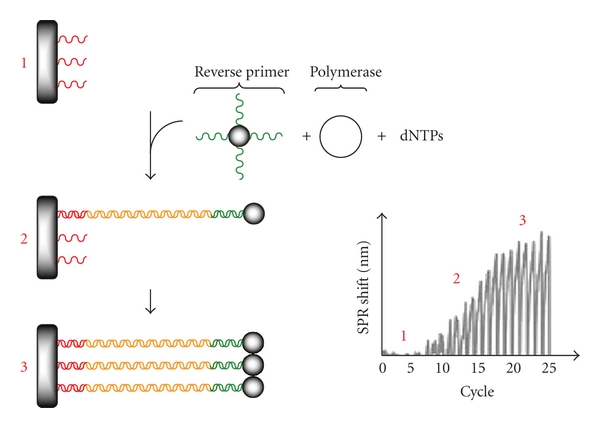
The amplification process results in an increasing amount of PCR product immobilized on the gold surface that can be detected as a change in the shift of the SPR wavelength [95].

Pollet et al. performed real-time monitoring of the amplification of an 80 base pair oligonucleotide by combining solid-phase PCR and FO-SPR. In this first proof-of-concept report, the authors immobilized 5′ thiol modified forward primer on the optical fiber and used standard PCR conditions (Taq polymerase, dNTPs, etc.). Due to the negative impact on the overall performance caused by adsorption of the polymerase on the gold surface, mercaptoalkane compounds were immobilized, to prevent the nonspecific interactions of the enzyme. Moreover, the sensitivity was increased by linking the reverse primer to gold nanoparticles (cf.  Figure 10). Pollet et al. were able to determine the efficiency of the solid-phase amplification. Compared to other reports of solid-phase PCR, the efficiency was similar (20–30%). In conclusion, the authors described an innovative new readout mechanism for real-time PCR using SPR.

### 5.12. Ritzefeld et al., Minor Groove Recognition Is Important for the Transcription Factor PhoB: A Surface Plasmon Resonance Study, 2011 [96]

Recently, we analyzed the interaction between the DNA-binding domain of the bacterial transcription factor PhoB (PhoB^DBD^) and its cognate DNA sequence (*pho* box) by SPR. We immobilized biotinylated 18 and 24 bp dsDNA molecules that contain the entire or parts of the *pho* box of the regulon *pst* on a streptavidin surface. In addition to the wildtype PhoB^DBD^ protein, two point mutants were used as analyte, where amino acids involved in the DNA recognition process were substituted by alanine. In spite of a systematic optimization (e.g., oligonucleotide length, surface concentration), an evaluation of the kinetic data using numerical integration did not result in a reliable fit. Therefore, the equilibrium dissociation constants were elucidated using nonlinear regression to fit the response units in equilibrium at different analyte concentrations to a one-site binding model. In consideration of circular dichroism results of the DNA-protein complexes, the SPR data revealed new insights into the binding mechanism of PhoB^DBD^.

Comparing the *pho* box sequences of different regulons that only differ in the minor groove additionally proved the dependency of the DNA-protein interaction on the groove composition. Beside the width of the corresponding minor groove, the bending properties of the DNA molecule and certain interactions mediated by amino acid residues have to be considered.

## Abbreviations

BAT: Biotin-terminated thiol
Biotin-11-UTP: Biotin-11-2′-deoxyuridine 5′-triphosphate
BirA: *E. coli *biotin ligase
CCD: Charge-coupled device
DMT: Dimethoxytrityl
DNA: Deoxyribonucleic acid
dsDNA: Double stranded DNA
DTT: Dithiothreitol
*E. coli*: *Escherichia coli*
EDC: Ethyl-*N*′-(dimethylaminopropyl)-carbodiimide
EDTA: Ethylenediaminetetraacetic acid
EMCH: *N*-[*ε*-Maleimidocaproic acid]-hydrazide
FO-SPR: Fiber-optic-based surface plasmon resonance
hAGT: O^6^-alkylguanine-DNA-alkyltransferase
IFC: Integrated microfluidic cartridge
IHF: Integration host factor
MEG: Maleimide-ethylene glycol-terminated disulfide
MUAM: Fmoc-protected 11-mercaptoundecylamine
NHS: *N*-hydroxyl succinimide
NTA: Nitrilotriacetic acid
OEG: Oligo(ethylene glycol)-terminated thiol
PCR: Polymerase chain reaction
PDEA: Pyridinyldithioethanamine
PNA: Peptide nucleic acid
RNA: Ribonucleic acid
RNase H: Ribonuclease H
RU: Response units
SAM: Self-assembled monolayer
SBP: Streptavidin binding peptide
SELDI: Surface-enhanced laser desorption/ionization
SPFS: Surface plasmon resonance fluorescence spectroscopy
SPR: Surface plasmon resonance
SPRi: Surface plasmon resonance imaging
SrtA: Sortase A
ssDNA: Single stranded DNA
SSMCC: Sulfosuccinimidyl 4-(N-maleimidomethyl) cyclohexane-1-carboxylate
ssRNA: Single stranded RNA
Sulfo-GMBS: *N*-[*γ*-maleimidobutyryloxy] sulfo-succinimide ester
TdT: Terminal Deoxynucleotidyl Transferase
TIR: Total internal reflection.
